# Intraoperative Epicardial Triventricular Pacing in a Pediatric Patient

**DOI:** 10.19102/icrm.2019.101205

**Published:** 2019-12-15

**Authors:** Johannes C. von Alvensleben, Marco A. Pinder, Caitlin Brateng, Max Mitchell, Kathryn K. Collins

**Affiliations:** ^1^Department of Pediatrics, Division of Cardiology, University of Colorado, Children’s Hospital Colorado, Aurora, CO, USA; ^2^Department of Surgery, Division of Cardiac Surgery, University of Colorado, Children’s Hospital Colorado Aurora, CO, USA

**Keywords:** Pacemaker, pediatrics, resynchronization

## Abstract

Cardiac resynchronization therapy (CRT) is used as an adjunctive therapy in adults with advanced heart failure but remains less commonly applied in pediatric patients. Further, CRT is traditionally conducted via biventricular transvenous pacing from the right ventricle and coronary sinus to activate the left ventricle and improve electromechanical synchrony; however, triventricular pacing, in which a third ventricular lead is utilized to activate an additional ventricular location, has been shown to be a feasible therapeutic alternative to typical CRT in patients with advanced heart failure or nonresponders. Limited adult studies involving triventricular pacing have been performed to date but no pediatric data are available. Thus, we present the case of a 12-month-old patient with congenital complete heart block and subsequent pacemaker-induced cardiomyopathy in whom triventricular epicardial pacing was applied in an effort to increase the available knowledge.

## Introduction

Cardiac resynchronization therapy (CRT) using biventricular pacing is a well-established adjunctive treatment in adult patients with advanced heart failure.^1,2^ Traditional CRT consists of pacing from the right ventricle and coronary sinus, with the aim of correcting electrical dyssynchrony/delayed activation of the lateral left ventricular (LV) wall. Studies in children and those with congenital heart disease are similarly promising but more limited in number.^3,4^ Although CRT can performed via the transvenous approach in this population, an epicardial approach is often required in these patients secondary to their small size or limitations in vascular access. This technique involves placing ventricular leads on the diaphragmatic surface of the heart with the atrial leads positioned on either the right or the left atrium—whichever affords the best pacing and sensing thresholds. The ventricular leads are manipulated to ensure optimal electromechanical synchrony.

More recently, triventricular pacing has emerged to improve outcomes of CRT “nonresponders,” frequently estimated to be 30% of adult patients.^2^ The use of an additional ventricular lead has been shown to improve electromechanical synchrony and acute echocardiographic (ECG) parameters.^5^ Although triventricular pacing is becoming more common in adult patients, there are, to our knowledge, no reported cases of such in pediatric patients. We herein present a pediatric patient with pacemaker-induced cardiomyopathy in whom epicardial triventricular pacing maneuvers were trialed and which resulted in improved acute intraoperative ECG function and QRS duration.

## Background

The patient was a 12-month-old male with prenatally diagnosed congenital complete heart block and neonatal lupus secondary to maternal anti-Ro antibodies. The postnatal average ventricular heart rate was 50 bpm, with a QRS duration of 81 ms. ECG revealed normal ventricular function, but the patient had mild endocardial fibroelastosis of the papillary muscles. He underwent placement of a dual-chamber epicardial pacing system involving atrial and ventricular leads (model 4968; Medtronic, Minneapolis, MN, USA) on the second day of life. The cathode of the ventricular lead was placed at the LV apex, with the anode at the ventricular septum. Normal ventricular size and function were documented prior to discharge. No endocardial fibroelastosis was observed in subsequent ECGs. Electrocardiography during pacing revealed a right bundle branch morphology with a QRS duration of 94 ms.

At nine months of age, the patient presented with increased labored breathing, decreased oral intake, and lethargy. ECG demonstrated severe LV dilation with a globular appearance and severely decreased function (ejection fraction: 14.8%). The paced QRS duration was 125 ms, with a narrow complex underlying the junctional escape rhythm at 70 bpm. The infant was found to have profound metabolic acidosis requiring intubation and epinephrine and milrinone infusions, and a decision was made to transition to VVI at 60 bpm to promote a narrower QRS and reduced ventricular pacing, with subsequent rapid weaning off of his inotropic drips (of epinephrine and milrinone). Ventricular function improved to moderately depressed (ejection fraction: 38%) with persistent moderate to severe dilation. The patient was discharged home, requiring ventricular pacing 40% of the time. He continued to struggle with poor weight gain despite caloric optimization and afterload reduction. ECG revealed unchanged moderately decreased function and moderate to severe LV dilation. In light of this, the decision was made to move forward with CRT.

### Procedure

**Figure 1:**
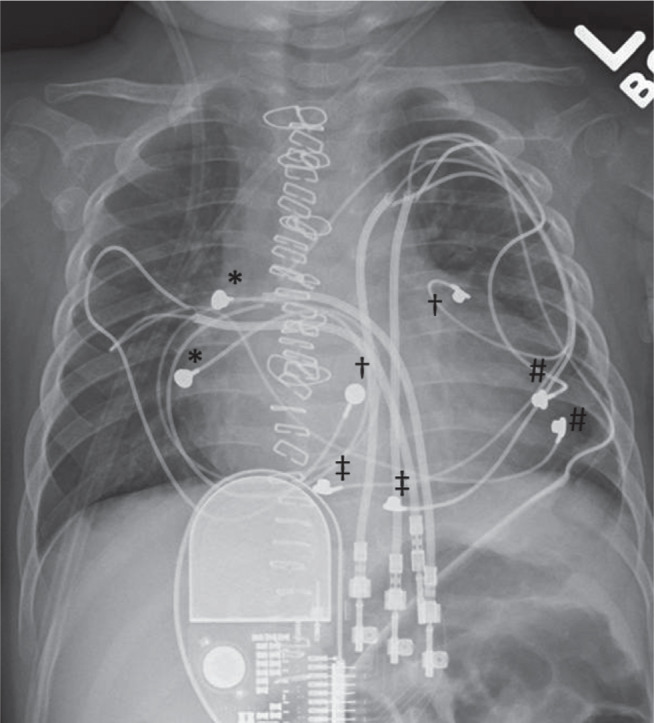
Chest radiograph demonstrating the epicardial pacing system. ^*^Bipolar atrial lead (chronic lead); ^#^bipolar LV apical lead (chronic lead); ^†^bipolar RV lead; ^‡^bipolar LV basolateral lead.

The patient was taken to the operating room, where a new model 4968 lead (Medtronic, Minneapolis, MN, USA) was placed on the diaphragmatic surface of the right ventricle (RV) near the atrioventricular groove. While pacing from this lead, the points of latest LV activation were sought and a new model 4968 lead (Medtronic, Minneapolis, MN, USA) was then placed at the base of the LV near the obtuse marginal branch of the left coronary artery. This location additionally achieved near-maximal distance between pacing ventricular pacing sites. The original LV apical lead was unchanged **(Figure 1)**.

The two LV leads were paired together using a twin bipolar-to-bipolar connector (2XBIS/BIS-17; Oscor Inc., Palm Harbor, FL, USA). Pacing was performed via the analyzer, contrasting biventricular and triventricular pacing with comparisons of QRS duration. Multiple configurations and pacing intervals were tested with a shortest QRS duration of 90 ms being achieved with biventricular pacing, utilizing either simultaneous RV and lateral LV pacing or lateral LV-to-RV pacing with a 30-ms delay **(Table 1)**. There was no significant difference in QRS duration between biventricular and triventricular pacing. There was an improvement in acute subjective ventricular function with similar ejection fraction values seen with both maneuvers, although the overall impedance was significantly lower when utilizing the triventricular pacing site (520 Ω versus 926 Ω). Given that biventricular pacing had not been trialed in this patient with similar acute results to triventricular pacing and there was a concern for battery longevity with the lower impedance, the decision was made to proceed with biventricular pacing. The chronic atrial lead and new ventricular leads were connected to a St. Jude PM3222 CRT device (Abbott Laboratories, Chicago, IL, USA) and the chronic LV ventricular lead was capped and placed into the pacemaker pocket. The patient tolerated the procedure well and was discharged to home on the fourth day after surgery.

Throughout a follow-up period of 32 months, repeat ECGs demonstrated normalization of the ventricular size and function.

## Discussion

CRT has become a common adjunctive therapy in adult patients with advanced heart failure and intraventricular conduction delay, resulting in improved ventricular electromechanical synchrony, positive remodeling, and reduced mortality.^2^ Despite these successes, approximately one-third of adult patients who undergo CRT device implantation fail to show any improvement in clinical symptoms and/or ECG evidence of positive remodeling. The addition of a third ventricular lead has been proposed and studied in adult patients,^5–8^ suggesting improvement in functional measures (eg, ejection fraction, ventricular volume, six-minute walk time, and Minnesota Living with Heart Failure score) as well as a reduction in all-cause mortality and the need for transplant. These results appeared to be true either when utilizing multiple LV pacing sites^5,7,8^ or RV pacing sites.^6^ The long-term complications (eg, lead dislodgement, device infection, refractory phrenic nerve capture) were comparable between the biventricular and triventricular groups, but there was a trend of shorter batter life noted in the triventricular group.^8^ This shorter battery life is believed to be secondary to the lower overall impedance that results from the existence of multiple lead contact points.

Of note, there are limited data available with regard to even traditional CRT application in pediatric patients.^3,4^ In a multicenter retrospective review, Dubin et al. examined the short-term safety and efficacy of biventricular CRT in pediatric patients and those with congenital heart disease.^3^ The majority of patients experienced an improvement in ventricular ejection fraction and shortening of the QRS duration when CRT was initiated, with those with congenital complete heart block and subsequent pacemaker-induced cardiomyopathy being most likely to respond.

The present case illustrates that triventricular pacing is feasible from an epicardial position by utilizing a bipolar-to-bipolar connector to simultaneously activate two LV positions. This pacing configuration affords the use of right and left basilar locations, which have been shown to be advantageous in pediatric epicardial biventricular pacing,^4^ while also providing left apex pacing, which is preferable for epicardial single-site ventricular pacing.^9^ The acute measurements were essentially unchanged between the two pacing modalities (biventricular versus triventricular), which is consistent with findings in adult studies, while long-term triventricular pacing was not trialed secondary to concerns of battery longevity. By leaving the capped left apical lead in the pacemaker pocket, conversion to chronic triventricular pacing would have been straightforward to pursue if biventricular pacing had not induced a positive response.

## Conclusion

Triventricular pacing can be feasible in a pediatric patient by utilizing epicardial leads and a bipolar-to-bipolar connector. The acute intraoperative ECG and electrical parameters are similar to those of biventricular pacing, although long-term pacing was not performed. The literature available on adult subjects suggests that triventricular pacing may provide an alternative therapy for nonresponders to biventricular pacing, but additional studies, including especially those involving pediatric patients, are required.

## Figures and Tables

**Table 1:** Association Between Pacing Maneuvers and QRS Durations

Pacing Maneuver	QRS Duration
Intrinsic QRS	86 ms
Simultaneous LV lateral + RV pacing	90 ms
LV lateral → RV 30-ms delay	90 ms
LV lateral → RV 20-ms delay	92 ms
LV lateral + LV apex + simultaneous RV	96 ms
LV apex → RV 20-ms delay	107 ms
LV apex pacing only	172 ms

LV: left ventricle/ventricular; RV: right ventricle/ventricular.
